# Early adversity in rural India impacts the brain networks underlying visual working memory

**DOI:** 10.1111/desc.12822

**Published:** 2019-03-21

**Authors:** Sobanawartiny Wijeakumar, Aarti Kumar, Lourdes M. Delgado Reyes, Madhuri Tiwari, John P. Spencer

**Affiliations:** ^1^ School of Psychology University of Stirling Stirling UK; ^2^ Community Empowerment Lab Uttar Pradesh Lucknow India; ^3^ School of Psychology University of East Anglia Norwich UK

**Keywords:** fNIRS, India, preferential looking, visual working memory

## Abstract

There is a growing need to understand the global impact of poverty on early brain and behavioural development, particularly with regard to key cognitive processes that emerge in early development. Although the impact of adversity on brain development can trap children in an intergenerational cycle of poverty, the massive potential for brain plasticity is also a source of hope: reliable, accessible, culturally agnostic methods to assess early brain development in low resource settings might be used to measure the impact of early adversity, identify infants for timely intervention and guide the development and monitor the effectiveness of early interventions. Visual working memory (VWM) is an early marker of cognitive capacity that has been assessed reliably in early infancy and is predictive of later academic achievement in Western countries. Here, we localized the functional brain networks that underlie VWM in early development in rural India using a portable neuroimaging system, and we assessed the impact of adversity on these brain networks. We recorded functional brain activity as young children aged 4–48 months performed a VWM task. Brain imaging results revealed localized activation in the frontal cortex, replicating findings from a Midwestern US sample. Critically, children from families with low maternal education and income showed weaker brain activity and poorer distractor suppression in canonical working memory areas in the left frontal cortex. Implications of this work are far‐reaching: it is now cost‐effective to localize functional brain networks in early development in low‐resource settings, paving the way for novel intervention and assessment methods.

**RESEARCH HIGHLIGHTS**

- The findings reveal the first reported impact of early adversity in rural India on the functional brain networks that underlie visual working memory (VWM).
- Children from higher socioeconomic status families showed a greater ability to detect change in a VWM task.
- Greater ability to detect change was associated with greater suppression of activation in the left frontal cortex.
- Activation in the left frontal cortex was associated with maternal education and income.

## 1 INTRODUCTION

Each year, 250 million children in low and middle income countries fail to reach their developmental potential (Black et al., 2017); Nowhere is this more critical than in Uttar Pradesh (UP), which is the most populous region in India (two‐thirds the population of the U.S.). UP, amongst the worst human development indicators, is socioculturally similar to other developmentally impoverished regions across South Asia, and holds a key to meeting global sustainable development goals. Quantitative analyses suggest that quality early intervention in such areas could be a very useful strategy in both social justice and economic terms (Hackman, Gallop, Evans, & Farah, 2015).

Poverty and early adversities significantly impact brain development, accentuating the risk of poor socioeconomic outcomes and contributing to a vicious cycle of poverty. Associations between socioeconomic status (SES) and early stunting and behavioural measures of cognitive and executive function have been well established (Fernald, Kariger, Hidrobo, & Gertler, 2012; Hackman & Farah, 2009). SES has also been found to correlate with executive functions across different tasks (Ardila, Rosselli, Matute, & Guajardo, 2005; Lipina, Martelli, Vuelta, & Colombo, 2005; Lipina, Martelli, Vuelta, Injoque‐Ricle, & Colombo, 2004; Mezzacappa, 2004; Sarsour et al., 2011).

Magnetic resonance imaging has shown that the development of brain structures through stages of early development is linked to SES, family income and parental education. Lower family income is associated with smaller sized frontal lobes (Hanson et al., 2013). By the age of 4, brains of lower SES children demonstrate lower grey matter in the frontal and parietal cortices (Hanson et al., 2013). Parental education has also been associated with prefrontal cortical thickness (Lawson, Duda, Avants, Wu, & Farah, 2013), total brain surface area (Noble et al., 2015) and volume of the hippocampus (Hanson et al., 2013; Noble et al., 2015) and amygdala (Noble et al., 2012). Similar relationships have been demonstrated between SES, income and education and white matter properties (Chiang et al., 2011; Gianaros, Marsland, Sheu, Erickson, & Verstynen, 2013).

Only a handful of studies have investigated how these demographic variables are related to brain function. Closely examining brain function is important because it can be an early and specific marker of emerging cognitive capabilities and provide direct insights into interventions. A previous study using event‐related potentials (ERPs) has shown that higher SES was associated with activation in the left frontal cortex (Tomarken, Dichter, Garber, & Simien, 2004). Further, ERP studies have shown that the amplitude of early components related to voluntary attention and detection of novelty arising from the frontal cortex is reduced in children of low SES families (Mezzacappa, 2004). A study utilizing functional magnetic resonance imaging (fMRI) has also shown that low SES children demonstrated an inefficient utilization of neural resources by activating parts of the frontal cortex that were not linked to behavioural improvements (Sheridan, Sarsour, Jutte, D'Esposito, & Boyce, 2012). However, it is harder to engage young children with cognitive tasks in an fMRI scanner because they would be required to stay completely still in a dark and noisy environment.

In our study, we used a low‐cost alternative to fMRI—functional near infrared spectroscopy (fNIRS)—to localize functional brain activation in young children in rural settings in India. fNIRS systems shine near‐infrared light into cortical tissue via sources placed on the head. Light is preferentially absorbed by oxyhaemoglobin (HbO) and de‐oxyhaemoglobin (HbR), scattered through tissue and some of the light is detected back at the surface by detectors placed near the sources. Previous work has demonstrated that fNIRS can be used in low resource settings: Lloyd‐Fox and colleagues took an fNIRS system into a field station in Keneba in the Gambia to collect brain imaging data from infants and children (Begus et al., 2016; Lloyd‐Fox et al., 2017, 2014). They measured localized brain activation to visual and auditory social cues across the first 2 years of life. Using these brain measurements, they showed that within the first two months of life infants selectively responded to non‐social auditory stimulation. However, after 4–8 months, infants start to show a preference for social stimuli. These were the first studies to have demonstrated the potential for using fNIRS in a rural setting.

In the present study, we focused on a key cognitive system, visual working memory (VWM). VWM is a cognitive system that is used roughly 10,000 times per day for at least two kinds of work—comparison of percepts that cannot be viewed at the same time and detecting changes in the world when they occur (Vogel & Luck, 1997; Vogel, Woodman, & Luck, 2001). Behavioural studies have shown that the development of VWM begins in infancy and gradually improves throughout childhood and adolescence (Fitch, Smith, Guillory, & Kaldy, 2016). Due to its early emergence and the availability of tasks that can be used in infancy to predict later achievement (Rose, Feldman, & Jankowski, 2012), VWM is an excellent marker of early cognitive development. Moreover, neuroimaging studies have revealed that parts of a fronto‐parieto‐temporal network are involved in VWM processing as early as 3 years of age (Buss, Fox, Boas, & Spencer, 2014; Perlman, Huppert, & Luna, 2016). Critically, the frontal cortex is thought to play an important role in modulating function in the posterior cortex to enhance WM representations and/or suppress irrelevant information (Buss et al., 2014; Buss & Spencer, 2017).

In the present study, we used a portable fNIRS system along with an innovative analysis pipeline to extend previous work. We optimized the cap geometry a priori to cover regions of interest, thereby, economically using the small number of channels available (Wijeakumar, Spencer, Bohache, Boas, & Magnotta, 2015). We also used an image reconstruction method to move from conventional channel‐based fNIRS analyses on the surface of the head to voxel‐based analyses within the brain volume (Wijeakumar, Huppert, Magnotta, Buss, & Spencer, 2017). Critically, this method accounts for variance in optode placement, which can be considerable in fNIRS studies conducted across the world given large variations in head circumference (e.g. stunting).

In the current study, we examined how children in rural settings in UP (referred henceforth as Rural UP) performed on the VWM task, and whether this task is sensitive to the impact of early adversity. We also investigated whether the functional brain networks that underlie VWM in rural India overlap with functional brain networks measured in a Midwestern US sample of children. Finally, we examined the association between demographic variables that index early adversity and brain activation in the Rural UP sample.

## 2 METHODS

### 2.1 Participants

Data collection was carried out at the Community Empowerment Lab Field office in Shivgarh, UP, India. The field office was established in 2003 in the rural Shivgarh block (population ~105K) of Raebareli district, UP. Since then, the Shivgarh site has served as a model for community‐led research, and hosted several high‐quality studies, including large cohort studies and cluster randomized controlled trials (participants > 25K). The site has served as a global gold standard demographic surveillance site for the Grand Challenges including 12 studies on population metrics. The human development and nutrition indices of the site are similar to the average UP index. The population is predominantly rural, and largely engaged in small‐scale semi subsistence farming activities.

Participants were recruited from the villages around the Shivgarh field office. Families were brought from their villages to this facility. Forty‐two infants and children within the age range of 4–48 months participated. All participants were born full term. None of the participants or their mothers had been diagnosed with any major psychiatric illnesses or had unusual characteristics as observed by the research staff. Informed consent was obtained from the parents of all children and in compliance with research standards for human research set by the Institutional Review Board. All procedures were in accordance with the Helsinki declaration.

Data from 8 participants were excluded from the final analyses because they failed to contribute data to all working memory loads (*N* = 2), they lacked neuroimaging data across trials due to poor signal‐to‐noise ratio (*N* = 1), and experimenter error (*N* = 5). No participants were excluded because of failure to engage with the task. Thus, data from 34 participants (20 males) were included in the analyses. Table 1 reports age and demographic information from these included participants.

**Table 1:** Demographic information from included participants (*N* = 34)

Variables	*N*	Mean	*SD*	Range
Age in months				
4–9 months (<1 year old)	10	5.8	1.2	
12–24 months (1–2 year old)	9	19.6	2.0	
24–36 months (2–3 year old)	9	30.3	1.8	
36–48 months (3–4 year old)	6	42.8	3.9	
Maternal age	34	9,326	1710.4	6,667–14,685
Paternal age	34	10,638.6	1659.6341	8,030–16,060
Family members	34	5.912	2.8324	2–14
Children under 5 yo	34	1.324	0.5349	1–3
Income	34	76,088.235	52,199.1337	25,000–200,000
Maternal education	34	7.941	4.6	0–17
Paternal education	34	9.4	3.5436	4–17

In the sections below, we compare aspects of our findings to data from a study of VWM using the same task and fNIRS methods conducted in the Midwestern United States. According to the 2010 census, the racial composition of this Midwestern town was 82.5% white, 5.8% African American, 6.9% Asian, 0.2% Native American, 2% other races and 2.5% two or more races. 4.81% of the population over the age of 25 had less than a high school diploma, while 12.91% graduated from high school, and 82.27% had some college experience through to a graduate degree. The average income in this population was $40,722. The children in the Midwestern U.S. study were grouped into three age groups: 4 month olds (*N* = 16, *M* = 3.9 months, *SD* = 0.4 months, 7 girls), 1 year olds (*N* = 19, *M = *14.7 months, *SD* = 1.6 months, 10 girls) and 2 year olds (*N* = 22, *M = *26.2 months, *SD* = 1 month, 12 girls).

### 2.2 Demographic variables

Data were collected on parental education, income, caste, religion, number of family members and children, type of family setting in the household and home economic status through variables such as material of roof, floor and walls, electricity and agricultural and vehicular assets. SES scores were calculated by combining education, occupation and income using the revised Kuppuswamy and Prasad scale (Shaikh & Pathak, 2017).

### 2.3 Experimental task

**Figure 1:**
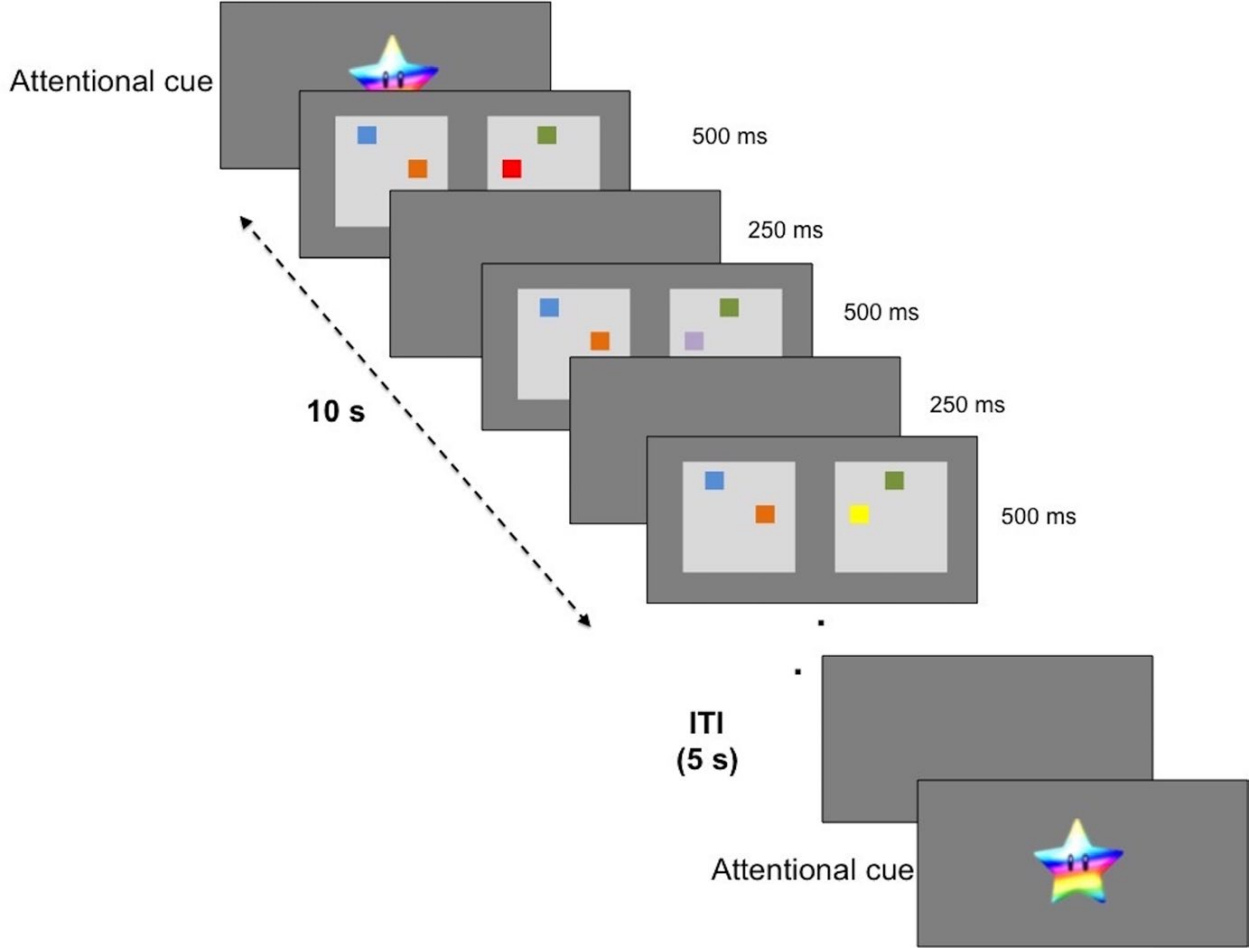
Schematic of the visual working memory (VWM) task

The VWM task is shown in Figure 1. An attentional cue was displayed until the child looked at the cue. As soon as the experimenter noted that the child was attending to the attentional cue, the experimenter began the trial with a key press. Each trial lasted for a duration of 10 s. During this period, two flashing displays of coloured squares were presented side by side. The squares were on the screen for 500 ms and then off for 250 ms. On one side, one randomly selected square changed its colour after each blink, while the colours of the other squares remained the same. Each trial was followed by a grey screen (inter‐trial interval) for 5 s. Each square in the display subtended a visual angle of 0.2 degrees. Previous studies have shown that infants around 13 months of age show above‐chance change preference (CP) scores at loads 1, 2 and 3, while older children (3‐ to 5‐year‐olds) show above‐chance CP scores at loads 2, 4 and 6 (Buss, Ross‐Sheehy, & Reynolds, 2018; Ross‐Sheehy, Oakes, & Luck, 2003; Simmering, 2016). Thus, we varied the VWM load between 1, 2 and 3 items (1, 2 or 3 squares on each side) for infants within the first 2 years of life, and we varied load between 2, 4 and 6 items (Simmering, 2016) for children who were older. Across all ages, these loads were classified as low, medium and high.

**Figure 2:**
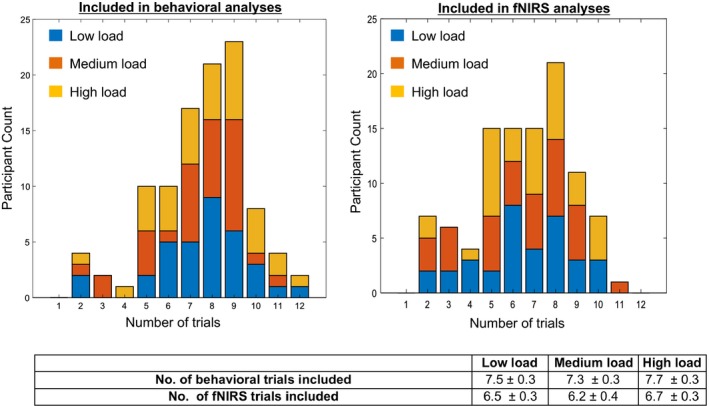
Histograms showing the participant count and trial count (bins) per load in the behavioural (left) and functional near infrared spectroscopy (fNIRS) (right) analyses

The experiment was created and presented using Director version 2.1. The experiment consisted of one run with 12 trials per load for each of the three loads. The total number of trials completed varied across participants. Participants were included if they contributed at least 1 trial per load. Figure 2 shows histograms of the number of trials contributed per participant for each load in the behavioural and fNIRS analyses. The number of included behavioural trials for the low load was 7.5 ± 0.3 trials, medium load was 7.3 ± 0.3 trials and high load was 7.7 ± 0.3 trials. The number of included fNIRS trials for the low load was 6.5 ± 0.3 trials, medium load was 7.2 ± 0.4 trials and high load was 6.7 ± 0.3 trials. We ran a repeated‐measures ANOVA with number of trials per load as a within‐subjects factor and age and gender as covariates to examine if participant age and/or gender might be associated with the number of trials they contributed. The number of trials contributed per load did not vary significantly by age or gender.

### 2.4 Procedure

**Figure 3:**
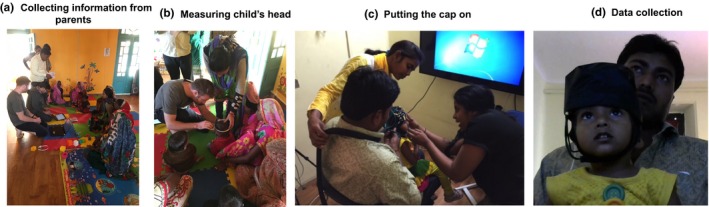
Overview of the data collection protocol

Participants with their families were welcomed and seated in a common playroom, where testing procedures were explained and consent was obtained. Demographic information about the families and children were also collected (Figure 3a). Children's head circumference was measured to prepare the fNIRS cap (Figure 3b). Then, the parent and child were accompanied to the room where testing would be carried out. The fNIRS research team was composed of 3 researchers—the first researcher was responsible for coding infants’ looking data, the second researcher was responsible for checking fNIRS signals and the third researcher was responsible for attending the child's and parent's needs. All three researchers were able to communicate with parents and children in Hindi and/or the local dialect of Awadhi. The cap was fitted onto the child's head (Figure 3c). In cases where hair needed to be moved under the NIRS sensors, two researchers moved hair with a hairpin to allow scalp contact. Throughout this process, children were soothed by engaging in conversation from the researchers and parents and/or shown cartoons on a laptop. We allowed the child to continue to watch cartoons during the process of digitizing the scalp landmarks and sources and detectors. Once the parent and child were comfortably seated, the experiment was begun and behavioural and brain‐imaging data were collected (Figure 3d).

### 2.5 Behavioural analyses

Looking data was manually coded by assigning each look to a ‘left’ or ‘right’ category based on whether the infant was looking at the left or right set of squares on the screen. Looks were not coded if the infant looked away from the screen or looked at the centre of the screen. Total looking time (TLT) for each trial was calculated as the total time spent looking at the left and right set of squares. Mean look duration (MLD) for each trial was calculated as TLT divided by the total number of looks. Switch rate (SR) was estimated as the number of switches from one side to the other within a trial divided by TLT and multiplied by 1,000 (to convert to seconds). CP was calculated as the looking time to the changing side divided by the TLT.

Statistical analyses were carried out on each of the behavioural variables using SPSS 22. Linear regression using the forward method was used to determine whether demographic variables predicted key behavioural measures in the VWM task. An outlier estimation of mean ± three times the standard deviation was applied to check if each regression was significant even after outliers were removed. All regression and correlation analyses included in the main text met this outlier check.

### 2.6 NIRS recording and processing

*Instrumentation.* A TechEn compact, wearable fNIRS machine (4 sources and 6 detectors) with wavelengths of 690 nm and 830 nm was used to HbO and HbR data from the infants and children. Light was transported from the machine to a customized cap geometry using fibre optics. The probe geometry consisted of 4 channels in each of the frontal hemispheres (Figure 3a). A part of this geometry was optimized for VWM studies by using regions of interest from the fMRI literature on VWM processing (Wijeakumar et al., 2015). Further, the same geometry was used to study changes in VWM processing in infants and children from a Midwestern US sample.

#### 2.6.1 Pre‐processing of NIRS data

HOMER2 (www.nmr.mgh.harvard.edu/PMI/resources/homer2) was used to pre‐process fNIRS data. Raw data were converted from intensity values to optical density units. Targeted principal components analysis was used to correct motion artefacts (Yücel, Selb, Cooper, & Boas, 2014). Then, the motionArtifactByChannel function (tMotion = 1.0, tMask = 1.0, StdevThresh = 50 and AmpThresh = 0.67) was used to detect any remaining segments with motion artefacts. We used the *StimRejection* function to reject trials that fell within noisy segments of data. These data were then band‐pass filtered to include frequencies between 0.016 and 0.5 Hz to remove respiration and cardiac rhythms. Data were then converted to concentration units using the modified Beer‐Lambert law. A general linear model was run on the HbO and HbR data separately with three regressors (low, medium and high loads). The onset of each of the included trials in each regressor was convolved with a modified gamma function of the form (exp(1) × (t – τ)^2^/σ^2^)*exp(−(tHRF − τ)^2^/σ^2^), where τ_HbO_ = 0.1, σ_HbO_ = 3, T_HbO_ = 10, and τ_HbR_ = 1.8, σ_HbR_ = 3 and T_HbR_ = 10. We used an ordinary least squares method to solve the GLM to obtain parameter estimates (Ye, Tak, Jang, Jung, & Jang, 2009). Thus, a beta estimate was obtained for each regressor (condition), channel, chromophore and participant.

#### 2.6.2 Forward model

Age‐specific atlases from the Neurodevelopmental MRI database (Richards, Sanchez, Phillips‐Meek, & Xie, 2016; Richards & Xie, 2015) were used to estimate the forward model. A 4–5 month‐old atlas was used for children who were between 4 and 9 months of age. A 1 year‐old atlas was used for children were between 12 and 24 months of age. A 2 year‐old atlas was used for children who were between 24 and 36 months of age. A 3 year‐old atlas was used for children who were between 36 and 48 months of age. Each age‐specific atlas was segmented into volumes for grey matter, white matter, cerebrospinal fluid and scalp tissue using 3dSeg from analysis of functional neuroimaging. 3D surface meshes were created from these tissue types using *iso2mesh*. Scalp landmarks and positions of sources and detectors were digitized using a Polhemus Patriot Sensor for most participants. For those participants that could not be digitized due to unavailability, we used points belonging to participants that had a similar head size (*N* = 5).

**Figure 4:**
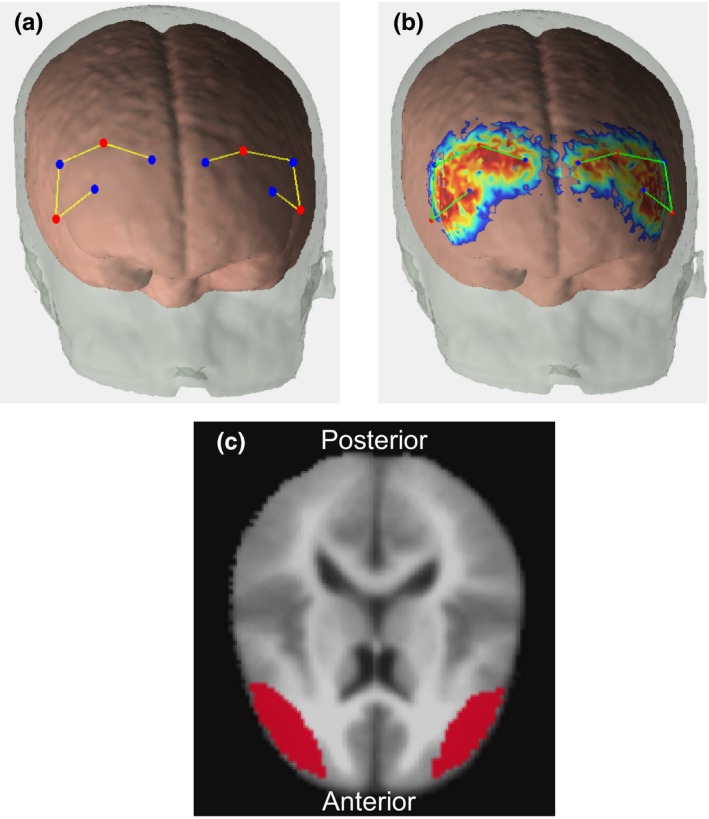
(a) Functional near infrared spectroscopy (fNIRS) probe geometry covering the frontal cortex on a single participant. Dots in red indicate sources and dots in blue indicate detectors. (b) Photon migration paths from running Monte Carlo simulations on a single participant. (c) Intersection mask of voxels containing data from at least 75% of the participants

Digitized points were projected onto the age‐specific atlases (Figure 4a) and Monte Carlo simulations with 100 million photons were run to create sensitivity profiles for each channel for each participant using *AtlasViewerGUI* (Figure 4b). The head volumes and sensitivity profiles were converted to NIFTI format. Participants’ sensitivity profiles were summed together, thresholded at an optical density value of 0.0001 (Wijeakumar et al., 2015) and transformed to MNI space to create subject‐specific masks. Subject‐specific masks from each age were summed together to create age‐specific masks. Within each of these age‐specific masks, only those voxels that contained data from at least 75% of the participants were taken forward to final analyses. Finally, all thresholded age‐specific masks were combined to create an intersection mask (Figure 4c).

#### 2.6.3 Image reconstruction

The methods for image reconstruction have been discussed in previous work (Putt, Wijeakumar, Franciscus, & Spencer, 2017; Wijeakumar, Huppert et al., 2017; Wijeakumar, Magnotta, & Spencer, 2017). Briefly, after accommodating for the forward model and beta coefficients (obtained from section 2.4.2), the relationship between the haemodynamic response and delta optical density is given by:

$$\left[\begin{array}{c} d.\varepsilon_{\text{HbO}}^{\lambda 1}.\beta_{\text{HbO}}+d.\varepsilon_{\text{HbR}}^{\lambda 1}.\beta_{\text{HbR}}\\ d.\varepsilon_{\text{HbO}}^{\lambda 2}.\beta_{\text{HbO}}+d.\varepsilon_{\text{HbR}}^{\lambda 2}.\beta_{\text{HbR}} \end{array}\right]=\left[\begin{array}{cc} \varepsilon_{\text{HbO}}^{\lambda 1}.F^{\lambda 1} & \varepsilon_{\text{HbR}}^{\lambda 1}.F^{\lambda 1}\\ \varepsilon_{\text{HbO}}^{\lambda 2}.F^{\lambda 2} & \varepsilon_{\text{HbR}}^{\lambda 2}.F^{\lambda 2} \end{array}\right].\left[\begin{array}{c} \Delta \text{Hb}{\text{O}}_{\text{vox}}\\ \Delta \text{Hb}{\text{R}}_{\text{vox}} \end{array}\right]$$

where, *d* is source‐detector distance, *F* is the channel‐wise sensitivity volumes from the Monte Carlo simulations. *Δ*HbO_vox_ and* Δ*HbR_vox_ are voxel‐wise relative changes in HbO and HbR concentrations and need to be estimated using an image reconstruction approach. We can re‐write this equation as:

Y=L.X

where,

$$Y=\left[\begin{array}{c} \beta_{\text{dOD}}^{\lambda 1}\\ \beta_{\text{dOD}}^{\lambda 2} \end{array}\right],\;L=\left[\begin{array}{cc} \varepsilon_{\text{HbO}}^{\lambda 1}.F^{\lambda 1} & \varepsilon_{\text{HbR}}^{\lambda 1}.F^{\lambda 1}\\ \varepsilon_{\text{HbR}}^{\lambda 2}.F^{\lambda 2} & \varepsilon_{\text{HbR}}^{\lambda 2}.F^{\lambda 2} \end{array}\right]\;\text{and}\;X=\left[\begin{array}{c} \Delta \text{Hb}{\text{O}}_{\text{vox}}\\ \Delta \text{Hb}{\text{R}}_{\text{vox}} \end{array}\right]$$

To solve for *X*, we used Tikhonov regularization and the system in the above equation can be replaced by a ‘regularized’ system given by,

$$X={\left(L^{T}L+\lambda .I\right)}^{-1}L^{T}.Y$$

where *λ* is a regularization parameter that determines the amount of regularization and *I* is the identity operator. Minimizing the cost function and solving for *X* can obtain voxel‐wise maps of relative changes in concentration for each condition, channel, participant and chromophore.

#### 2.6.4 fNIRS group analyses

A group‐level ANOVA was run on the voxel‐wise maps. The ANOVA had two factors: load (low, medium and high) and chromophore (HbO, HbR). Age was used as a covariate. The main effects and interactions from the ANOVA were corrected for family‐wise errors using 3dClustSim (corrected at α = 0.05, corresponding to a cluster size threshold of 21 voxels with a voxel resolution of 2 × 2 × 2 mm). Only effects that included a chromophore main effect or interaction are discussed. HbO and HbR concentrations are typically anti‐correlated, with HbO positive (and HbR negative), indicating an activation effect or HbO negative (and HbR positive), indicating a suppression effect. By only including significant chromophore effects, we focused on clusters with canonical haemodynamic signatures of neural activation. To verify this was the case at the individual participant level, we examined the beta values from each participant and each brain cluster that showed an association with behaviour or SES variables. We found that 65.2% of participants showed these canonical patterns between chromophores.

Voxels from the main effect and interactions were classified such that they belonged to only a single effect. Priority was given in the following order: (1) interaction between chromophore, load and age, (2) interaction between chromophore and load, (3) interaction between chromophore and age and (4) main effect of chromophore. Pearson's correlation was used to correlate behavioural and brain activation in these clusters. In addition, linear regression was used to examine the relationship between demographic variables and brain activation. An outlier estimation of mean ± three times the standard deviation was applied to check if each regression was significant even after outliers were removed. All regression analyses included in the main text met this criterion. Correlations and regressions were not corrected for multiple comparisons.

## 3 RESULTS

### 3.1 Behavioural results

Separate one‐factor ANOVAs were run with load as a within‐subjects factor to investigate how behavioural looking dynamics were associated with load [TLT (low load = 6,280 ± 1674 ms; medium load = 6,561 ± 1612 ms; high load = 6,616 ± 1652 ms), MLD (low load = 1814 ± 831 ms; medium load = 2,119 ± 906 ms; high load = 2,181 ± 982 ms) and SR (low load = 0.50 ± 0.18; medium load = 0.45 ± 0.19; high load = 0.44 ± 0.18)]. The main effect of load was significant for TLT and MLD (TLT: *F*_(2,63)_ = 3.416, *p* < 0.05, ƞ^2^ = 0.094; MLD: *F*_(2,59)_ = 6.459, *p* < 0.005; ƞ^2^ = 0.164). However, only pairwise comparisons for MLD achieved significance after family‐wise corrections. Here, MLD at the high and medium loads was greater than MLD at the low load (*p* < 0.05).

**Figure 5:**
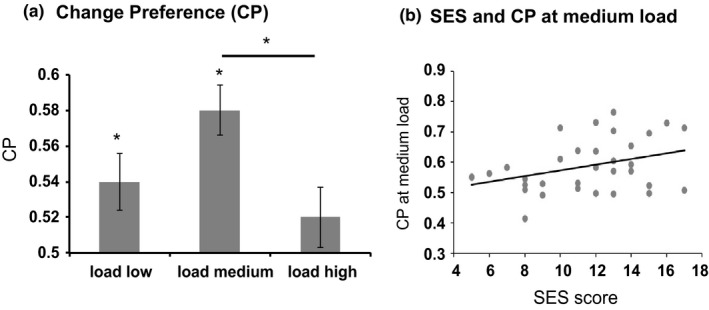
(a) Change preference (CP) scores at the low, medium and high loads. *indicate significant above‐chance performance. (b) Relationship between CP at the medium load and socio‐economic status (SES) score

A key measure in the VWM task is the CP score at each load (total looking to the change side/total looking to both displays). Above‐chance performance indicates that children are able to detect which display is changing and therefore, that the memory load was within their working memory capacity. Figure 5a shows CP scores at all three loads. As in previous studies (Ross‐Sheehy et al., 2003), we conducted one‐tailed *t*‐tests to compare CP scores at each of the loads against chance (0.50). CP scores at the low and medium loads were significantly greater than chance (*p* < 0.05, Cohen's *d* = 0.42 for low load and Cohen's *d* = 0.99 for medium load), replicating findings from US studies (Ross‐Sheehy et al., 2003). This suggests that the VWM task is culturally transferable.

Given that children performed their best at the medium load, we used CP scores at the medium load as an index of individual differences in the task. A step‐wise multiple linear regression was run to examine whether socioeconomic variables predicted CP at the medium load. SES scores significantly predicted CP at the medium load (*F*_(1,32)_ = 4.726, *p* < 0.05, *R^2^* = 0.129). Figure 5b shows the relationship between CP at the medium load and SES score: as SES increased, CP scores increased. Thus, the VWM task is sensitive to the impact of adversity on early cognitive development.

### 3.2 Brain activation results

To examine how brain activity varied as the working memory load increased, we conducted an ANOVA on the voxel‐wise fNIRS data with load (low, medium and high) and chromophore (HbO and HbR) as factors and age as a covariate. After family–wise corrections, significant clusters of activation emerged for the main effects of and interactions between load, age and chromophore. Table 2 presents the significant clusters, their coordinates and a summary of the association between HbO activation in these clusters and behavioural variables and demographic variables.

**Table 2:** Regions of interest and their relationship to behavioural and demographic variables. Regions that are discussed in the main text have been abbreviated here for ease of reference. ‘+’ and ‘−’ in the last two columns indicate positive and negative correlations respectively

Cluster	Hemi	Size (mm)	Centre of Mass			Correlation with behaviour	Predicted by demographic variables
			*x*	*y*	*z*		
Interaction between chromophore, age and load							
Inferior frontal gyrus/dorsolateral prefrontal cortex (rIFG/DLPFC)	R	2,512	−40.6	−38.7	7.4	CP at medium load (−)	
Dorsolateral prefrontal cortex (lDLPFC1)	L	264	40.2	−32.4	17.6		Maternal education (+)
Middle frontal gyrus (lMFG)	L	200	30.4	−44.6	35.8	MLD at medium load (+)	Income (−)
Interaction between chromophore and age							
Dorsolateral prefrontal cortex (lDLPFC2)	L	1,096	40.5	−30.7	26	CP at medium load (−)	
Superior frontal gyrus (rSFG)	R	176	−20.7	−39.7	36	MLD at low load (+) SR at low load (−)	
Dorsolateral prefrontal cortex	R	72	−41.6	−29.8	17.3		
Interaction between chromophore and load							
Middle frontal gyrus	L	472	27.9	−45.3	29.5		
Dorsolateral prefrontal cortex	L	192	44.2	−38.5	24.8		
Inferior frontal gyrus (lIFG1)	L	184	51	−35.1	−3.3		Income (+) Maternal education (+)
Chromophore main effect							
Inferior frontal gyrus	R	560	−46.1	−30.8	10.2		
Superior medial gyrus	R	280	−7.4	−57.1	30.7		
Superior frontal gyrus	L	280	12.1	−56.1	31.1		
Inferior frontal gyrus	L	208	51.6	−22.7	27.7		
Superior frontal gyrus	R	168	−20.2	−56.2	20.6		
Inferior frontal gyrus	R	40	−39.6	−33.2	2.4		
Inferior frontal gyrus	R	24	−42.7	−38.7	10		
Inferior frontal gyrus	R	16	−48	−30	1		
Dorsolateral prefrontal cortex	R	16	−40	−30	15		

MLD: mean look duration; SR: switch rate; CP: change preference.

**Figure 6:**
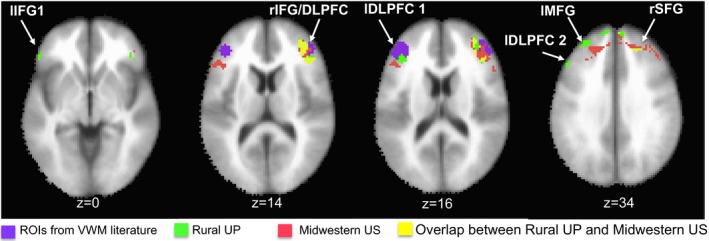
Spatial overlap in clusters with significant brain activation patterns across the Rural UP and Midwestern US samples

To examine whether functional brain activation in the Rural UP sample was localized to the canonical VWM network, we pooled all significant clusters and spatially overlapped them with significant effects from a comparable study of a Midwestern US sample (Reyes, Wijeakumar, Magnotta, & Spencer, [Link]). Figure 6 shows the spatial overlap between effects from the Rural UP and Midwestern US samples. There was robust overlap in the right inferior frontal gyrus/dorsolateral prefrontal cortex/ (rIFG/DLPFC; shown in yellow in Figure 6) and right superior frontal gyrus (rSFG; shown in yellow in Figure 6), but also unique activation across both samples (shown in green for Rural UP and in red for Midwestern US).

**Figure 7:**
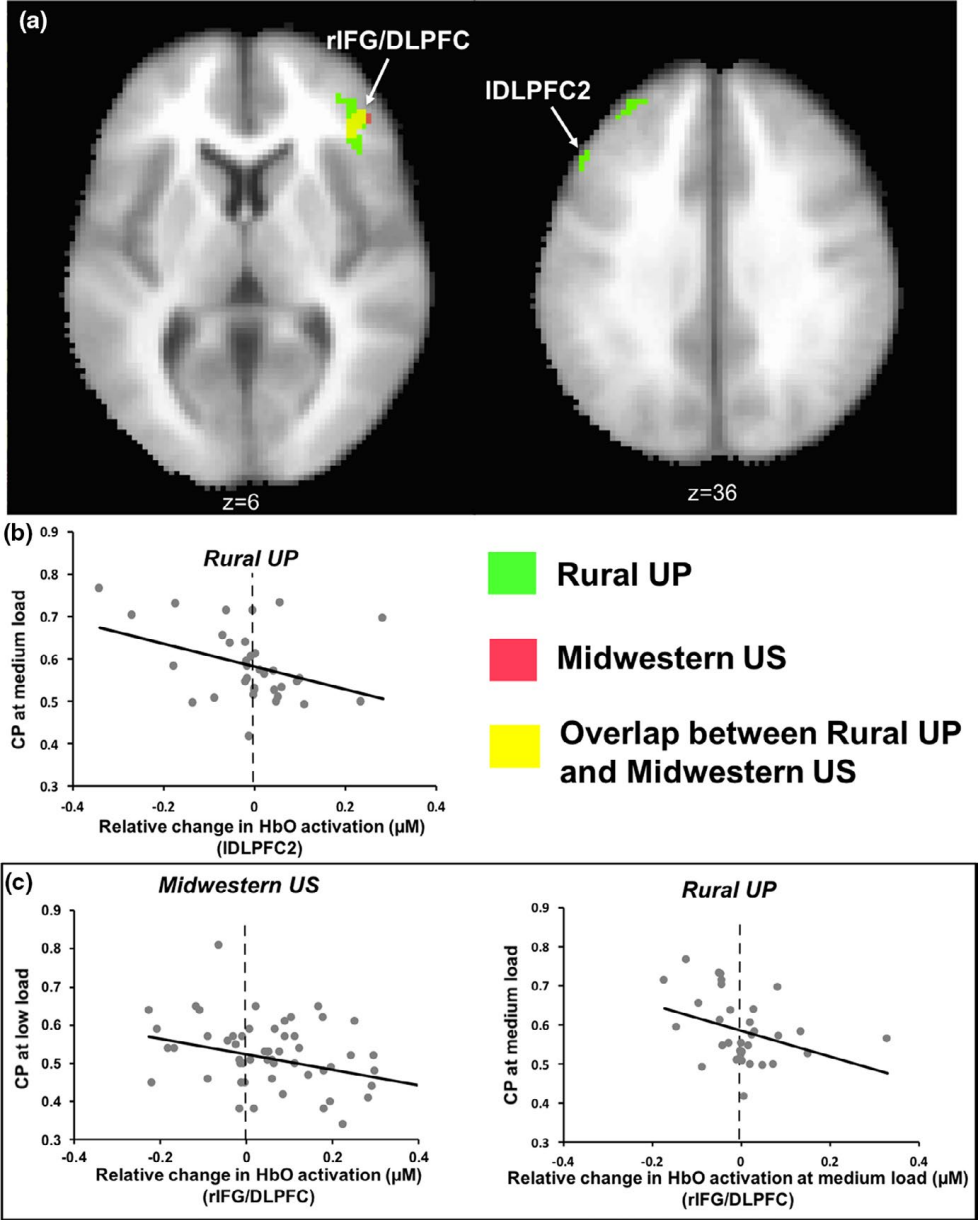
(a) Areas where oxyhaemoglobin (HbO) activation is negatively correlated with change preference (CP) at the medium load. (b) Negative correlation between HbO activation in lDLPFC2 cluster and CP at the medium load. (c) Negative correlation between HbO activation in rIFG/DLPFC and CP at the medium load. Both the Rural UP and Midwestern US samples show this trend in the same cluster (shown in yellow in a)

Spatial overlap across samples is one index of how brain activity is similar, but are overlapping brain regions performing the same function? Here, we chose all clusters of HbO brain activation from Table 2 and correlated brain activation in each cluster with behavioural measures (e.g., CP scores) using Pearson's correlation. Two clusters showed a negative correlation between HbO brain activity and CP at the medium load (see Figure 7a): rIFG/DLPFC (Figure 7c: *r *= −0.41, *p* < 0.02; *xyz* coordinates −40.6, −38.7, 7.4) and lDLPFC2 (Figure 7b: *r *= −0.42, *p* < 0.02; *xyz* coordinates 40.5, −30.7, 26). In these clusters, reduced activation was associated with a greater ability to detect change. Of these, the rIFG/DLPFC cluster also overlapped with a cluster from the Midwestern US sample that showed the same negative relationship with CP scores (shown in Figure 7c). In VWM tasks, the frontal cortex is thought to regulate parietal and temporal cortices to drive attention to and maintain stimulus representations (Edin et al., 2009; Wijeakumar, Magnotta et al., 2017; Zanto, Rubens, Thangavel, & Gazzaley, 2011). The frontal cortex also plays a role in suppressing distracting information (Cosman, Lowe, Zinke, Woodman, & Schall, 2018). Our results may reflect distractor suppression in early development: children with higher CP scores showed a suppression of frontal activity, which may have prevented the non‐changing display from capturing attention.

**Figure 8:**
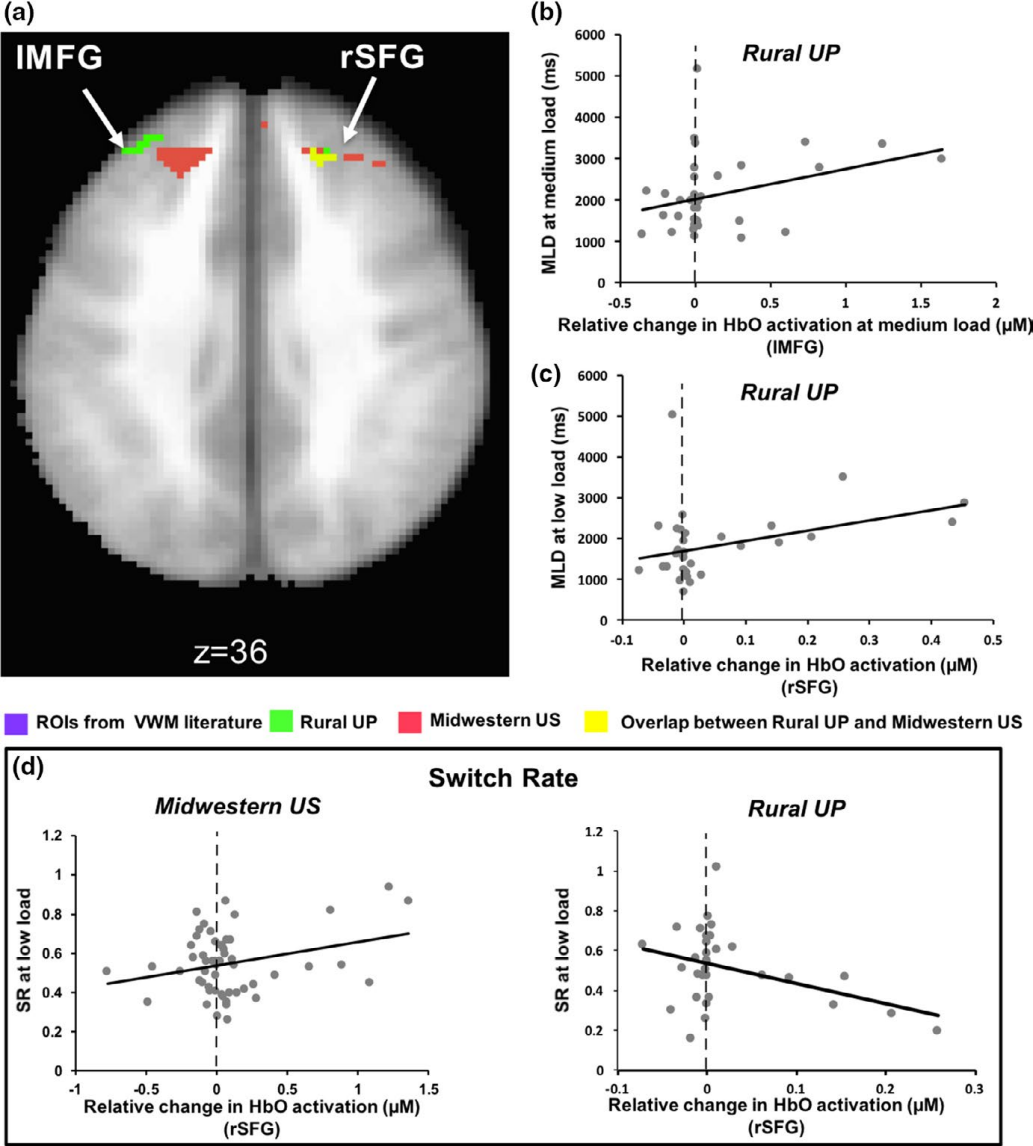
(a) Areas where oxyhaemoglobin (HbO) activation is correlated with mean look duration (MLD) and switch rate (SR). (b and c) Areas showing a positive correlation between HbO activation in lMFG and rSFG and MLD. (d) Negative correlation between HbO activation in rSFG and SR at low load in the Rural UP sample. On the other hand, HbO activation in the same cluster (shown in yellow in a) was positively correlated with SR at low load in the Midwestern US sample

In addition to brain‐behaviour correlations involving the primary behavioural measure (CP), we also conducted brain‐behaviour correlations with looking measures (MLD and SR; see Table 2). These correlations indicate how HbO activation in these clusters changed as a function of children's visual exploratory behaviour. There were two clusters that showed a significant correlation with visual exploration measures (MLD and/or SR; Table 2 and Figure 8a): lMFG with MLD (Figure 8b: *r = *0.41, *p* < 0.02; *xyz* coordinates 30.4, −44.6, 35.8) and rSFG with MLD (Figure 8c: *r* = 0.54, *p* < 0.002) and with SR (Figure 8d: *r = *−0.38, *p* < 0.03; *xyz* coordinates −20.7, −39.7, 36). In the lMFG cluster, greater HbO concentration was associated with greater dwell times. Interestingly, the rSFG cluster spatially overlapped with a cluster in the Midwestern US sample; however, in this case, the Midwestern US sample showed a positive correlation between SR and HbO concentration (shown in Figure 8d).

**Figure 9:**
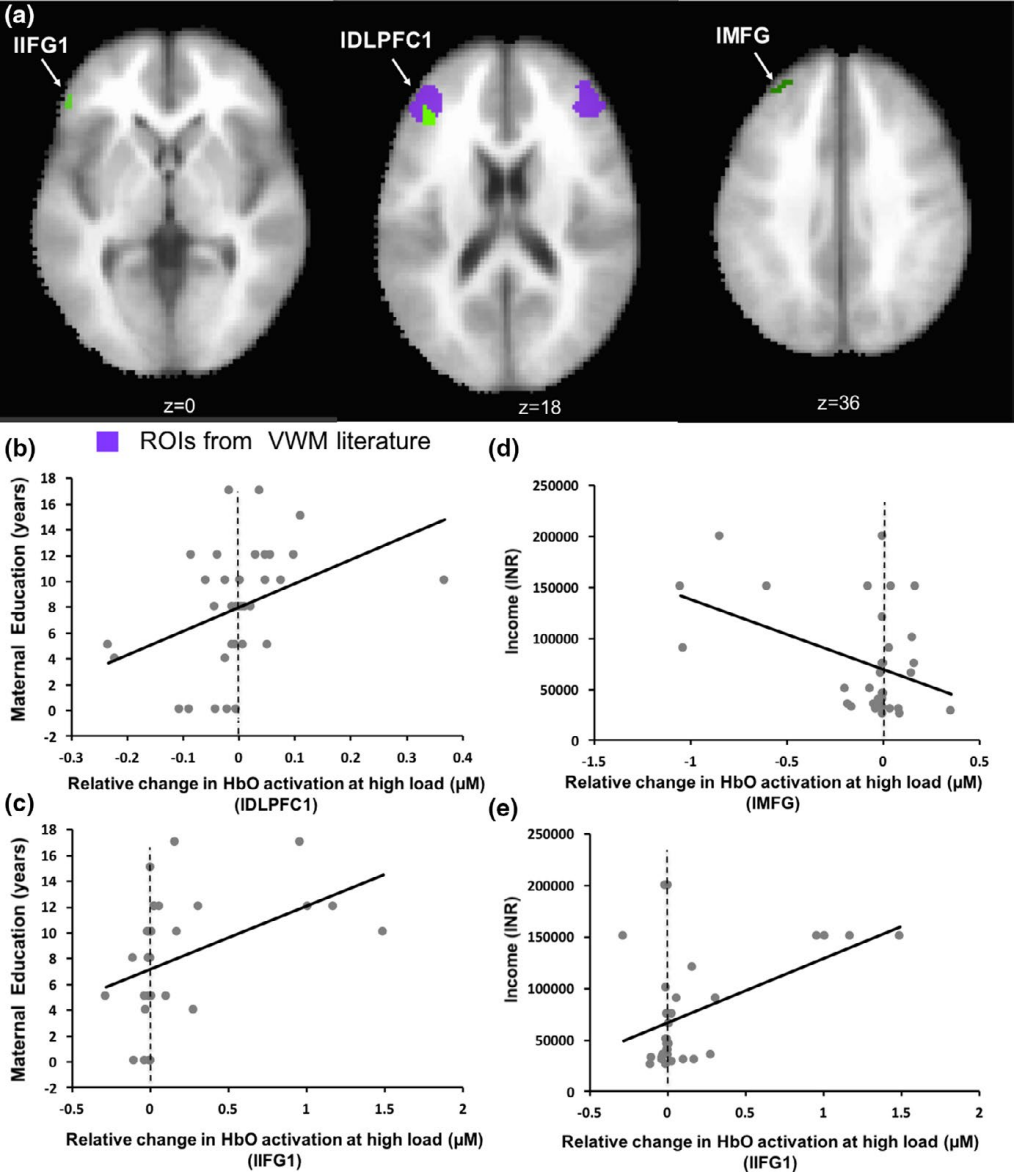
(a) Areas that show an association with socioeconomic variables. (b and c) Oxyhaemoglobin (HbO) activation in lDLPFC1 and lIFG1 are positively correlated with maternal education (d) Negative correlation between HbO activation in lMFG and income. (e) Positive correlation between HbO activation in lIFG1 and income

In a final analysis, we used linear regression to investigate whether HbO activation in the Rural UP sample (see clusters in Table 2 and Figure 9a) was predicted by demographic variables, that is, does functional brain activity show an impact of early adversity? Maternal education predicted HbO activation at the high load in lDLPFC1 (Figure 9b: *F*_(1,33)_ *= *5.594, *p* < 0.05, *R^2^ = *0.149; *xyz* coordinates 40.2, −32.4, 17.6) and lIFG1 (Figure 9c: *F*_(1,32)_ *= *6.387, *p* < 0.05, *R^2^ = *0.166; *xyz* coordinates 51, −35.1, −3.3). In both cases, greater maternal education was associated with greater left frontal cortical activation. This is consistent with previous studies linking parental education with structural brain development (Hanson, Chandra, Wolfe, & Pollak, 2011; Noble et al., 2015). Interestingly, HbO activation at the high load in lIFG1 was also predicted by family income (Figure 9e: *F*_(1,33)_ = 9.226, *p* = 0.005, *R^2^* = −0.224; *xyz* coordinates 51, −35.1, −3.3). Thus, we used a stepwise linear regression with both income and maternal education as predictors to determine if one variable captured more variance. We found that income alone accounted for 22% of variance in HbO activation in lIFG1. HbO activation at the high load in lMFG was also predicted by family income (Figure 9d: *F*_(1,33)_ = 6.77, *p* < 0.05, *R^2^* = −0.175; *xyz* coordinates 30.4, −44.6, 35.8).

## 4 DISCUSSION

A key scientific challenge is to understand the impact of early adversity on functional brain development. Here, we report findings from the first functional neuroimaging study in rural India that examined the impact of early adversity on neurobehavioural correlates of VWM. Our data demonstrate that a portable neuroimaging technology and innovative analytical methodology can be combined to investigate behavioural and brain activation during early development in a rural setting.

Infants and children engaged in the VWM task, successfully detecting the changing side at low and medium loads. This suggests that the VWM task is transculturally transferable. We identified CP at the medium load as a key behavioural index for VWM processing. Critically, children who belonged to high SES families were able to detect change at this load better than children who belonged to low SES families. Studies from other countries have shown associations between SES and behavioural measures that are comparable to our findings. Lipina et al. (2005) reported that, on average, infants from low SES families were less advanced in WM performance and inhibitory control in the A not‐B task. Farah and colleagues assessed multiple prefrontal cognitive systems through a series of behavioural tasks. They found that cognitive ability was not affected across all the tasks in children from low SES backgrounds, but, rather, disparities in SES affected WM, cognitive control, language and memory (Farah et al., 2006). Our results are not in agreement with a more recent study by Ursache and Noble (2016) who found an association between SES factors and cognitive flexibility, but not WM or inhibitory control. However, they used a list sorting working memory test, which might have engaged long‐term memory processes rather than isolating WM.

Localized, task‐specific activity in the frontal cortex of children from this Rural UP sample showed remarkable spatial overlap with a Midwestern US sample demonstrating robust cross‐cultural engagement of the frontal VWM network. These findings demonstrate that our image‐based fNIRS approach can effectively target specific cortical sites even with few channels using a portable system operated in a remote site. Critically, the high spatial precision of the image‐based fNIRS approach allowed us to effectively align task‐specific neural responses across samples. This was most clearly demonstrated in the rIFG/DLPFC region. Here, both samples not only showed overlapping functional brain activation, but also demonstrated an inverse relationship with CP scores. In both cases, as VWM load increased, the activity in this region was suppressed. In VWM tasks, the frontal cortex is thought to regulate parietal and temporal cortices to drive attention to and maintain stimulus representations (Edin et al., 2009; Wijeakumar, Magnotta et al., 2017; Zanto et al., 2011). The frontal cortex also plays a key role in suppressing irrelevant or distracting information (Cosman et al., 2018). We assert that the suppression of the frontal cortex in the VWM task required that children sustain looking to the changing side and prevent the flashing display on the non‐changing side from capturing their attention.

We also found that suppression in the lDLPFC (lDLPFC2) was inversely correlated with CP scores in the Rural UP sample, showing more widespread suppression of frontal activity in children with higher CP scores. This is consistent with two studies. Stevens and colleagues conducted an auditory attention task and found that there were no SES differences in the ERP responses from children when they were asked to attend to a particular narrative. However, low SES children also showed an ERP response to the narrative they were not supposed to attend to, suggesting that these children had difficulty suppressing distracting information (Stevens, Lauinger, & Neville, 2009). Kishiyama, Boyce, Jimenez, Perry, and Knight (2009) found that children from low SES backgrounds showed a reduction in the amplitude of the N2 component when presented with discriminable stimuli amidst repetitive stimuli. This component is related to novelty detection and is thought to originate from the prefrontal cortex. A reduction in N2 amplitude might be due to the inability to suppress irrelevant distractor information and detect novelty.

Our findings also revealed that activation in the lDLPFC was positively associated with maternal education. This is consistent with previous studies linking parental education with structural brain matter (Hanson et al., 2011; Noble et al., 2015). It is hypothesized that more educated parents tend to spend more time with their children, use diverse and complex language and engage in productive turn‐taking practices that encourage the social well‐being of the child (Landry, Smith, & Swank, 2006). We are currently examining how Indian mothers with different educational backgrounds interact with young children during dyadic play sessions to uncover links between daily visual exploratory exchanges and performance in the VWM task.

Relatedly, children raised in families with greater income showed stronger activation in the lIFG cluster. Greater income enables the provision of better facilities in homes, better feasibility of time‐rich care‐giving, better opportunities for enhancement of cognitive abilities and learning and affordability of more items in homes that can create diverse learning environments for children. A study by Duncan, Morris, and Rodrigues (2011) showed that across 20,000 observations of children between 2 and 15 years of age, increased household income resulted in increased cognitive performance. Specifically, a $1,000 increase in annual family income led to better outcomes in math and reading skills, which have been robustly related to WM processing. Our findings also revealed a negative association between brain activation in a cluster in the lMFG and income; children belonging to families with lesser income showed greater activation in the lMFG cluster. Interestingly, greater activation in this cluster was also related to greater dwell time (MLD). Taken together, these findings suggest that children from lower income families might have trouble disengaging from visual displays when they need to.

Although there was robust overlap between the Rural UP and Midwestern US samples, there were also interesting differences. In particular, the Rural UP sample showed more widespread left frontal activation, while the Midwestern US sample showed more widespread right frontal activation. Critically, left‐lateralized activation in the Rural UP sample also reflected the impact of early adversity, showing robust associations with both parental education and income. We argue that adversity might have an impact on the left hemisphere. This is a particularly important observation because over development, this hemisphere tends to become specialized for working memory functions (Thomason et al., 2009). Note, however, that there were cross‐cultural differences in right hemisphere activation as well. In particular, greater activation in rSFG was associated with longer looks and a lower shift rate in the Rural UP sample, while greater activation in rSFG was associated with less mean looking and a higher shift rate in the Midwestern US sample. Thus, it is likely that differences across the samples reflect the operation of an integrated frontal network that spans both hemispheres.

The current study has several limitations. Portable systems have fewer numbers of channels and therefore, limit the amount of brain coverage. This limitation was partially counteracted by optimizing the cap geometry a priori; however, we were unable to record from parietal and temporal cortices involved in VWM (Wijeakumar et al., 2015). We also used age‐specific MRI atlases from western samples for image reconstruction. We acknowledge that there might be differences in morphological and volumetric features of head and brain templates of children from the rural UP and mid‐western US samples. Richards and colleagues found that Chinese age‐specific templates were shorter, wider and taller than US‐specific templates (Xie et al., 2015). Such differences between the two samples could have an effect on image reconstruction. In an ongoing project, we are currently collecting structural MRIs from rural UP to examine the effect of using age‐, culture‐ and SES‐specific templates on image reconstruction.

Finally, a few participants contributed only 2 trials per condition when one run was presented to them. While this is on the lower end of the number of trials per participant, it highlights the potential of the experimental task and neuroimaging technique to be used in field settings where multiple challenges might not favour long experimental runs.

**Figure 10:**
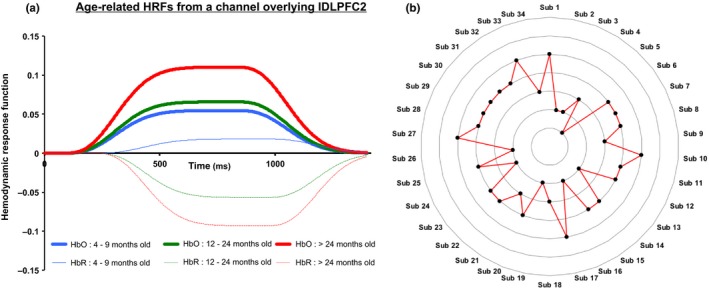
(a) Haemodynamic response functions (HRFs) for oxyhaemoglobin (HbO) and de‐oxyhaemoglobin (HbR) activation for three age groups obtained from a channel overlaying lDLPFC2. (b) Radar plot showing individual differences across the 34 participants. The seven rings represent seven significant effects from Table 2 (based on associations between HbO activation and behavioural/demographic variables). For instance, participants 1, 10, 17, 27 and 33 showed *ideal* activation patterns in five out of seven effects, whereas participant 5 showed *ideal* activation pattern in only one effect. Refer to main text for an elaborate description

Although we observed effects of SES variables on behavioural and brain data even with relatively sparse data in some cases, there are several ways in which future work could improve upon our methods. A first improvement would be to explicitly sample children from low and high SES backgrounds. We are doing this in an ongoing project, collecting behavioural and brain data from larger numbers of children from specific age groups and socioeconomic strata. Another potential improvement in methodology might be to use multiple sets of basis functions (Arichi et al., 2012; Aslin, Shukla, & Emberson, 2015) in the GLM to better capture age‐specific haemodynamic response functions (HRFs) instead of using the same canonical impulse response function across individuals. Differences in the time course data over ages were evident in some channels. For instance, in Figure 10a, we show time course data across ages from a channel overlying the lDLPFC2 cluster. There is a clear increase in amplitude and an apparent temporal shift as well, with earlier activation in the older children. With a larger sample across age groups, this could be examined in detail using multiple basis functions to capture variations in the haemodynamic time course more precisely.

Future work should also examine whether differences in the brain response across clusters is robust within individuals. To probe this issue with our sample, we classified HbO activation from seven significant effects in Table 2 (those showing associations with behavioural and demographic data) into *ideal* and *non‐ideal* activation patterns. In particular, we conducted a median split of the data for each effect. If participants’ data fell within the upper half of the distribution for an effect where greater brain activation was considered beneficial (activation in lMFG positively correlated with MLD), it was classified as an ‘ideal‘ pattern. Additionally, if participants’ data fell within the lower half of the distribution for an effect where supressed was considered beneficial (suppression in rIFG/DLPFC correlated with CP), it was classified as an ‘ideal’ pattern. Data that fell outwith these criteria were classified as ‘non‐ideal.’ In Figure 10b, we show a radar plots of the results: some participants show ideal activation in five out of the seven effects, whereas other participants show ideal activation in only one out of seven effects. It would be useful in future work to track children longitudinally to see whether a classification like this can be used as a biometric risk index.

Although our data have limitations, we overcame multiple challenges whilst collecting data from the cohort. They ranged from sociocultural issues such as engaging the children and their parents with the experimental protocol and methodological constraints such as the effect of dark hair on signal quality. We were able to tackle these issues by making sure that all the personnel in our team could communicate with each other and with the parents and children. Speaking the same language as the families minimized confusion and built trust between the research team and the families. Each team member was trained to work efficiently within their role, but was also aware of the duties of other members, so that they could step in to handle another duty if required. For example, researchers would actively interact with the children using toys and cartoons to soothe them when the cap was being fitted, even if this required researchers to change roles in the process. Finally, we ensured that each step in the experimental protocol was clearly communicated to the parents so they could help us as partners during the session.

In summary, our findings demonstrate that fNIRS is a sensitive assessment tool that can guide future efforts to overcome the impact of adversity on functional brain development. Most importantly, our results reveal the first reported impact of early adversity in rural India on the functional brain networks that underlie VWM. These data also demonstrate that a portable neuroimaging technology and innovative analysis methods can be applied in a rural, low resource setting to precisely measure the impact of early adversity on behavioural and brain development. This could have far‐reaching implications, bringing cutting edge technology to the settings in which it is most severely needed.

## CONFLICT OF INTEREST

The authors declare no competing financial interests.

## AUTHOR CONTRIBUTION

JPS, SW and AK developed the concept and design for the study. SW, LDR and JPS performed the experiment and carried out the analyses. SW, JPS and AK wrote the manuscript.
